# Detection of fragmentation while dusting during retrograde intrarenal laser lithotripsy: a novel computer vision and perception pipeline

**DOI:** 10.1007/s10103-026-04843-2

**Published:** 2026-03-05

**Authors:** Jonathan E. Katz, Orlando Diaz-Ramos, Christopher Yong-Zyn Lo, Jamie Finegan, Tung Yen Chiang, Yijie He, Zekai Liang, Michael Yip, Roger L. Sur, Shan Lin

**Affiliations:** Departments of Urology and Electrical/Computer Engineering, University of California, San Diego, USA

**Keywords:** Laser Lithotripsy, Computer Vision, Ureteroscopy

## Abstract

Dusting during ureteroscopy with laser lithotripsy is popular because it minimizes the need for basketing of fragments. However, one of the challenges with dusting is adjusting power settings to be efficient and to limit the inadvertent generation of fragments. Detection of fragment production in real time using computer vision could help facilitate energy adjustments to optimize dusting. With IRB approval, we recorded eight consecutive ureteroscopy with laser lithotripsy procedures performed for intrarenal stones with a single-use flexible ureteroscope. We used a Dornier Thulio laser to dust the stones, with power settings at the discretion of the surgeon. We included all patients with stones between 7 mm and 15 mm. Time and duration of clips showing fragmentation were labeled. We then developed a fragmentation during dusting detection pipeline based on contemporary AI technologies, including semantic segmentation and video point tracking, and qualitatively demonstrated the detection performance on paradigmatic video segments. Successful extraction was performed in all 8 surgical videos, but only 5 had sufficient fragmentation events and adequate visualization for inclusion. We evaluated our pipeline’s performance on frame-level fragmentation detection, achieving an F1 score of 0.568, driven by a recall (sensitivity) of 0.516 and a precision (positive predictive value) of 0.632. Segment-level fragmentation detection performance was substantially lower, with an F1 score of 0.124 due to low precision (0.072) despite a recall of 0.438. We also qualitatively analyzed our pipeline on representative segments and provide insight into the task-specific challenges. Recent advancements in video point tracking enable more accurate capture of object motion patterns, which could be used to track the fracture of fragments from the kidney stone. With refinement, the proposed pipeline for the detection of fragmentation may be utilized to provide feedback to the surgeon and/or laser to adjust power settings in real time.

## Introduction

 Ureteroscopy with laser lithotripsy (URS) is a minimally invasive procedure used to treat kidney stones up to 1.5 cm to 2 cm in size, depending on location [1]. The procedure involves inserting a ureteroscope into the kidney equipped with a laser to break up the kidney stone into small pieces. These pieces are either deemed small enough to pass on their own or are basketed out of the kidney.

Advancements in high power laser technologies have enabled urologists to apply lower energy settings with higher frequency to achieve efficient dusting capabilities [2]. However, different lasers and different stone types have different optimal laser settings [3] and to some extent each stone requires trial and error to optimize dusting efficiency while limiting inadvertent creation of fragments or undue retropulsion of stones. Both inadvertent fragmentation and excessive stone retropulsion can increase operative time and may require additional stone retrieval maneuvers, including basketing or suctioning.

Computer vision models have previously been utilized to localize stones in images derived from ureteroscopy footage [4–6] and could even identify the location of a fragment [4, 6]. However, when optimizing laser settings, it is specifically the real-time detection of fragmentation in video that is crucial. To our knowledge, this has not yet been successfully achieved.

Currently, many deep learning models for computer vision tasks typically require several thousand to even over 100,000 images for training [7] to ensure a certain level of robustness and generalizability. However, fragment data is relatively scarce and time-consuming to review and label. To address these challenges, we developed a novel method for identifying fragmentation using a transformer-based point tracking model. This architecture has been a key contributor to the recent success of foundation models in computer vision tasks, particularly in generalizing to previously unseen data [8]. Herein, we describe our method and results for the identification of inadvertent fragmentation during URS using a three-stage pipeline requiring: semantic segmentation to identify the kidney stone, video point tracking on the stone surface, and finally, point clustering to monitor for new fragment generation.

## Methods

### Patients

With approval from the Institutional Review Board, we recorded eight consecutive ureteroscopy with laser lithotripsy procedures performed with a Dornier Axis single-use flexible ureteroscope and a Dornier Thulio laser (a high-power pulsed Thulium: YAG laser). In all cases we intended to dust the stones with power settings at the discretion of a single surgeon. We included all patients with stones between 7 and 15 mm.

### Data collection

We then converted the videos to images at 30 frames per second and labeled time and duration of segments of fragment vs. dust formation. We defined dusting as stone ablation, where the resulting particles are small enough to float or approximately ≤ 250 μm [9] while fragmentation was identified when the resulting fragments appeared to be greater than 1000 μm in diameter to avoid equivocal cases for tiny fragments between 250 μm and 1000 μm. Videos were included if ≥ 5 fragmentation events were able to be labeled during the video.

### Modeling and analysis

**Fig. 1:**
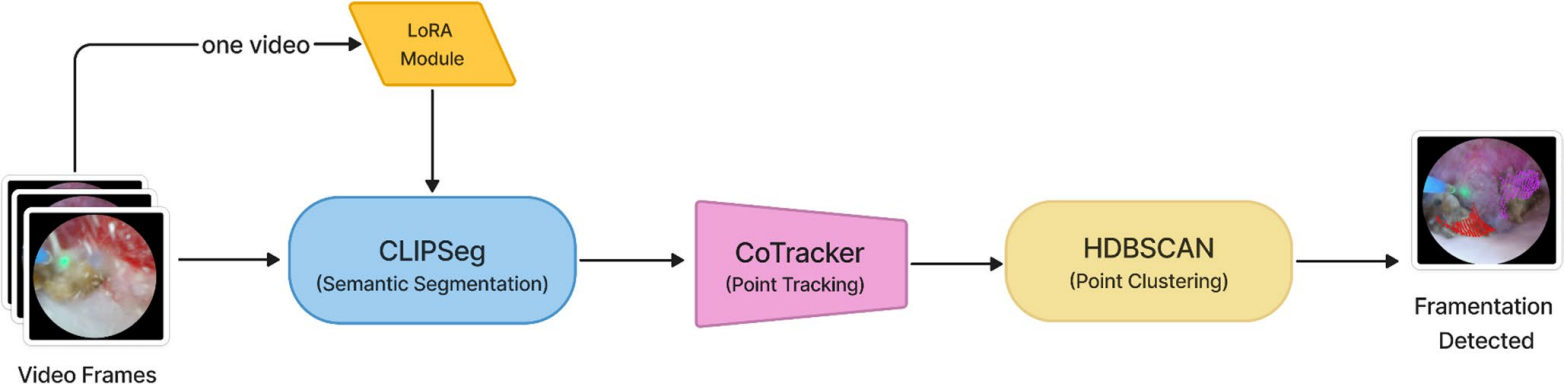
The image processing pipeline. Sequential video frames are passed into CLIPSeg and LoRA modules for identifying fragment pixels, CoTracker is then used to track the pixels over time, while HDBSCAN finds clusters of moving points to identify when a fracture happens and a fragmentation event occurs

We utilized a three-stage pipeline to detect the generation of fragments (Fig. 1). First, it conducts semantic segmentation to identify kidney stones (including fragments) from the video. Next, it tracks a group of points on the stone surfaces. It can then determine whether a fragment has broken off from the stone by checking if a group of points has split into two clusters based on point positions or their motion patterns, which we explain in more detail below.

Semantic segmentation involves classifying each pixel in an image into a predefined category, allowing us to find every pixel belonging to a kidney stone. To ensure the input mask is accurate, we evaluated several state-of-the-art segmentation models, including DeepLabV3 [10], U-Net [11], U-Net++ [12], and CLIPSeg [13], finding CLIPSeg provided the best segmentation quality. CLIPSeg combines is a foundation model for general image segmentation and required finetuning to segment novel classes. In this case, for ureteroscopy images, CLIPSeg was fine-tuned with Low-Rank Adaptation (LoRA) [14] using 142 annotated images, with 38 from Case 2 and 104 from Case 8.

Next, tracking points on the stone surface across a video sequence can capture the motion patterns of the stone and is required to identify the distinct motion pattern that occurs when a fragment breaks off. CoTracker [15] is the state-of-the-art pretrained model for dense point tracking built on the transformer architecture and trained over a broad range of image sequences [8]. It exhibited strong generalization ability on unseen input data, even on ureteroscopy videos, requiring no fine-tuning.

When a fragment breaks from the kidney stone, a subgroup of the tracked points will be separated from other points in terms of their positions and motions. By clustering points based on their positions and motion patterns, we can potentially determine whether the tracked points come from a single stone or from both a stone and several fragments that have broken off from it. We conduct clustering only based on point positions, and once the number of clusters increases, we predict that a fragment may have been generated. In this work we leveraged HDBSCAN [16], an advanced algorithm that automatically determines the number of clusters based on the density of data points given that we cannot know how many clusters/fragments appear in the simage. The method has been shown to be more robust to noise and varying cluster shapes, making it well-suited for the unpredictable nature of stone fragmentation. DBSCAN [17] is a related method, but HDBSCAN uses a hierarchical approach to improve robustness and reduce the need for manual parameter tuning.

### Outcomes

To quantify the performance of our algorithm, we analyzed the results both for single frames and for video segments (3 s [90 frames]). For individual frames, we considered a true positive (TP) if the predicted frame number fell within ± 30 frames (± 1 s) of any frame within a ground truth fragmentation event interval (defined by start and end frames). If a prediction matched a ground truth interval, all frames within that interval were counted as TPs for that specific interval, but only once, even if multiple predictions applied to the same interval. Similarly, if a ground truth interval has no matching prediction frame, all frames within that interval are counted as False Negative (FN). For segments, we counted 1 TP if any successful frame-level prediction occurred within that segment. If there was no labeled fragmentation in the period it was labeled a FP, and each missed labeled event was labeled as a FN. F1 scores were calculated to evaluate each model’s performance by calculating the harmonic mean of precision (or positive predictive value) and recall (sensitivity).

## Results

Successful extraction was performed in all 8 original videos, but only 5 surgical videos had sufficient fragmentation events and adequate visualization for inclusion. Overall, 137 fragmentation events were labeled. We evaluated the performance of our proposed three-stage pipeline (semantic segmentation with CLIPSeg, point tracking instability detection with CoTracker, and clustering with HDBSCAN) on these videos, named Case 2,3,6,7, and 8. Performance was assessed at both the frame-level (single frame) and segment-level (3-second segments [90 frames]).

The results indicate moderate frame-level recall (0.516), suggesting the model often detects activity near true fragmentation events. However, the corresponding frame-level precision (0.632) and particularly the high number of frame-level false positives point to a significant issue with false alarms (Table 1). This could result from the segment-level classification being determined by whether at least one of the 90 frames in a segment is classified as fragmentation. Given the moderate frame-level precision, it is difficult to avoid false positives across all 90 frames, leading to a significantly higher false positive rate compared to the frame level.

**Table 1:** Overall performance metrics across all evaluated cases. Precision and F1 calculated from provided TP, FP, FN counts

Metric level	Metric	Value	Total TP	Total FP	Total FN
Frame-Level	Recall	0.516	2085	1211	1959
Frame-Level	Precision	0.632	2085	1211	1959
Frame-Level	F1 Score	0.568	2085	1211	1959
Segment-Level	Recall	0.438	60	768	77
Segment-Level	Precision	0.072	60	768	77
Segment-Level	F1 Score	0.124	60	768	77

At the segment level, the model achieved moderate recall (0.438), meaning it detected fragmentation within the correct 3-second window reasonably often. However, the segment-level precision was very low (0.072), again driven by a high number of false positive segments, indicating the model frequently predicted fragmentation in segments where none occurred (Table 1). This could result from the segment-level classification being determined by whether at least one of the 90 frames in a segment is classified as fragmentation. Given the moderate frame-level precision, it is difficult to avoid false positives across all 90 frames, leading to a significantly higher false positive rate compared to the frame level.

Performance varied across individual cases. For instance, Case 3 showed the highest frame-level F1 score (0.634), while Case 7 had the lowest (0.280). However, the critical metrics reflecting temporal precision and avoidance of false alarms such as segment-level precision, remained consistently low across all cases (Table 2).

**Table 2:** Performance metrics of each case. Precision and F1 calculated from provided TP, FP, FN counts

Case	Frame-Level Precision	Frame-Level Recall	Frame-Level F1 Score	Segment-Level Precision	Segment-Level Recall	Segment-Level F1 Score
2	0.399	0.408	0.403	0.04	0.357	0.072
3	0.766	0.540	0.634	0.094	0.416	0.153
6	0.283	0.464	0.351	0.192	0.625	0.294
7	0.171	0.786	0.280	0.072	0.714	0.132
8	0.354	1,000	0.523	0.158	1.000	0.273

### Qualitative examples

**Fig. 2:**
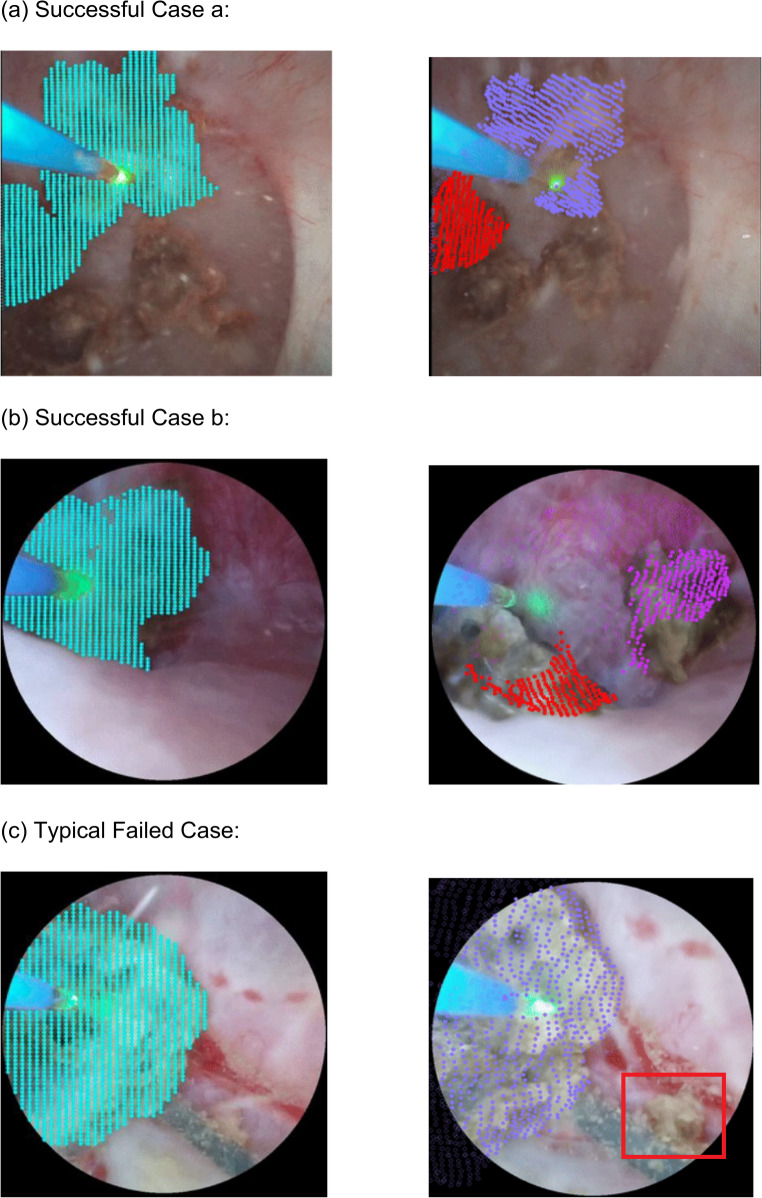
(**a**) Successful Case (**b**) Successful Case (**c)** Typical Failed Case

Below we show three typical events from our current detection results, where (a) and (b) are successful cases of fragmentation detection, while (c) is a failed case. The images on the left show how points to track are initialized by the semantic segmentation algorithm, while the images on the right show the point tracking and clustering results after a few seconds. If the clustering algorithm determines that the points are divided into two clusters, the points are visualized in two different colors. When the fragments are relatively large, the motion pattern of the points on the stone surfaces could be more accurately captured and clustered by the point tracking model as shown in Successful Case (a) and (b). While in (c), the fragment is much smaller than the whole kidney stone, and it moves fast after breaking off from the stone, so the point tracking model failed to capture it (Fig. 2).

## Discussion

The shift from human-controlled robotics to autonomous robotic surgery opens exciting frontiers for developing technologies to enhance surgical precision and improve patient outcomes. Data supports robotics offers enhanced precision, reduced morbidity and quicker return to recovery [18]. Therefore, the incorporation of iterative steps towards machine-automated endourology would be a natural evolution. One specific task in this field would be the development of an automated-assistant for the earliest detection of fragmentation while dusting during retrograde intrarenal laser lithotripsy.

Laser dusting offers significant advantages over traditional fragmenting in kidney stone surgery by reducing operative times up to 40% as it eliminates the need for active fragment retrieval and pulverizing stones into extremely fine fragments that can pass spontaneously in urine without requiring basket extraction. The dusting approach is associated with shorter procedure times and lower risk of ureteral damage compared to traditional fragmentation [2]. This technique also decreases stone retropulsion during the procedure, minimizing the need to chase migrated stones and potentially reducing postoperative complications.

One nuance of lasering is selecting the correct laser setting in order to ensure optimal dusting settings are used. Typically, dusting is achieved with low energy and high frequency (e.g. 0.4 J 20 Hz). Vendors will often recommend preset dusting settings but these are assumptions that do not always align with individual stones. Instead, the operator must toggle through settings until the optimal dusting setting is achieved. Herein, machine learning would seemingly have a role in helping to automate this step. Therefore, in this study, we developed a pipeline to facilitate autonomous fragment detection.

Our results demonstrated overall mixed performance, but performed better at the frame-level than segment level. We extracted images at 30 frames per second to match the native recording frame rate from the Dornier Axis ureteroscope. This proved critical because often fragmentation events might be brief - only 150 milliseconds (as few as 3–5 frames). This meant to adequately characterize moments of fragmentation we had to use a very high frame rate, which in turn led to much higher false negative rates. Similarly, while Lu et al., have had success with segmenting kidney stones and fragments, even during laser lithotripsy, small fragments, especially during ablation have remained a challenge [6]. Furthermore, we utilized a conservative fragment threshold of approximately 1 mm, however a more liberal threshold of two or three mm may have yielded improved results.

In both the frame and segment level analysis, our overall recall was moderate and suffered with respect to precision. This is to say that the algorithm was able to detect fragments with moderate sensitivity but also often overcalled fragments. The observed quantitative performance limitations, particularly the high false positive rate and low precision, likely stem from inherent challenges within each stage of our chosen pipeline and the complexity of the surgical video data itself. Firstly, the initial segmentation using general-purpose models like CLIPSeg may struggle with the specific visual artifacts of ureteroscopy, such as dust clouds, reflections, and low-resolution fragments, potentially leading to inaccurate point initialization for tracking. Secondly, CoTracker, designed primarily for consistent point tracking, is sensitive to occlusions, rapid motion, and domain shifts common in surgical videos; interpreting its instability as solely indicative of fragmentation is likely a major source of false positives. Finally, relying on HDBSCAN to detect fragmentation based purely on changes in the spatial clustering of points may be insufficient, as it doesn’t explicitly model the distinct motion patterns associated with a fragment breaking off and could misinterpret unrelated point cloud shifts. The limitations inherent in adapting general-purpose AI tools for this specific surgical application underscore the difficulty of achieving reliable fragmentation event detection with the current methodology.

In this way, future ureteroscopy specific vision models must overcome the unusual hurdles intrinsic to the procedure, such as that it is taking place in fluid, with large amounts of dust artifact, and sometimes bleeding. Furthermore, there can be significant movement with blur due to ureteroscope movement, patient respiration and/or pulsations from nearby vessels. While accurately labeling this data is challenging because the ground truth for any given image or clip may not even be known to the surgeon, we feel well labeled data is critical for fine-tuning general models for optimal performance in computer vision tasks.

Despite the challenges encountered, our study represents a novel investigation into the feasibility of detecting stone fragmentation events during ureteroscopy using a unique combination of contemporary AI technologies. Our approach diverges from traditional event detection methods by leveraging the instability characteristics of a point tracking foundation model, CoTracker, as a proxy signal for fragmentation. Specifically, we explored whether the disruption in tracking points, initialized via semantic segmentation (CLIPSeg) and identified through density-based clustering (HDBSCAN), could correlate with the physical event of a stone fragmenting. While the performance shows many limitations, this work contributes valuable insights into the application boundaries of these powerful general purpose models in complex, dynamic surgical environments and explores an unconventional methodology for event detection in a data-scarce domain, indicating the need for specifically designed models or fine-tuned methods for the unique challenges of more accurately analyzing fragmentation events in the future. Although our pipeline’s performance was modest, it can be applied to future laser lithotripsy videos to accelerate data labeling and support the creation of larger datasets for more robust training and improved fragmentation detection.

## Conclusion

Detection of fragment production is still preliminary using computer vision models. Recent advancements in video point tracking may enable more accurate capture of object motion patterns, which could be used to track the fracture of fragments from the kidney stone. With refinement, the proposed pipeline may be utilized to provide feedback to the surgeon and/or laser to adjust power settings in real time. 

## Data Availability

The datasets generated and analyzed during the current study are not publicly available due to the presence of Protected Health Information (PHI) and restrictions imposed by the Health Insurance Portability and Accountability Act (HIPAA). However, de-identified data may be available from the corresponding author upon reasonable request and subject to institutional approval and a formal Data Use Agreement (DUA).

## Abbreviations

AI: Artificial Intelligence
AUA: American Urological Association
CLIPSeg: Contrastive Language-Image Pretraining for Segmentation
CoTracker: Collaborative Transformer Tracker
CT: Computed Tomography
DBSCAN: Density-Based Spatial Clustering of Applications with Noise
F1 score: Harmonic Mean of Precision and Recall
FN: False Negative
FP: False Positive
FPS: Frames Per Second
HDBSCAN: Hierarchical Density-Based Spatial Clustering of Applications with Noise
IRB: Institutional Review Board
LoRA: Low-Rank Adaptation
TP: True Positive
URS: Ureteroscopy with Laser Lithotripsy
ViT: Vision Transformer
WCE: World Congress of Endourology

## References

[1] Assimos D, Krambeck A, Miller NL, Monga M, Murad MH, Nelson CP et al (2016) Surgical Management of Stones: American Urological Association/Endourological Society Guideline, PART I. J Urol 196(4):1153–1160 27238616 10.1016/j.juro.2016.05.090

[2] Matlaga BR, Chew B, Eisner B, Humphreys M, Knudsen B, Krambeck A et al (2018) Ureteroscopic Laser Lithotripsy: A Review of Dusting vs Fragmentation with Extraction. J Endourol 32(1):1–6 29061070 10.1089/end.2017.0641

[3] Johnson J, Lee J, Movassaghi M, Han D, Pingle S-R, Williams J et al (2024) Comparative Analyses and Ablation Efficiency of Thulium Fiber Laser by Stone Composition. J Urol 211(3):445–454 38134235 10.1097/JU.0000000000003833 PMC11292594

[4] Setia SA, Stoebner ZA, Floyd C, Lu D, Oguz I, Kavoussi NL (2023) Computer Vision Enabled Segmentation of Kidney Stones During Ureteroscopy and Laser Lithotripsy. J Endourol 37(4):495–501 36401503 10.1089/end.2022.0511

[5] Leng J, Liu J, Cheng G, Wang H, Quarrier S, Luo J et al (2024) Development of UroSAM: A Machine Learning Model to Automatically Identify Kidney Stone Composition from Endoscopic Video. J Endourol 38(8):748–754 38753704 10.1089/end.2023.0740

[6] Lu D, Deol ES, Koyama T, Oguz I, Kavoussi NL (2025) A computer vision model for automated kidney stone segmentation and evaluation of its performance vs surgeons. BJU Int 137(1):87-94 10.1111/bju.70001 PMC12690337 40994261

[7] Zhou T, Porikli F, Crandall DJ, Van Gool L, Wang W (2023) A survey on deep learning technique for video segmentation. IEEE Trans Pattern Anal Mach Intell 45(6):7099–7122 36449595 10.1109/TPAMI.2022.3225573

[8] Vaswani A, Shazeer N, Parmar N, Uszkoreit J, Jones L, Gomez AN et al (2017) Attention is all you need. arXiv [csCL]

[9] Keller EX, De Coninck V, Doizi S, Daudon M, Traxer O (2021) What is the exact definition of stone dust? An in vitro evaluation. World J Urol 39(1):187–194 32270283 10.1007/s00345-020-03178-z

[10] Chen L-C, Papandreou G, Schroff F, Adam H (2017) Rethinking Atrous Convolution for Semantic Image Segmentation. arXiv [csCV]

[11] Ronneberger O, Fischer P, Brox T (2015) U-Net: Convolutional Networks for Biomedical Image Segmentation. arXiv [csCV]

[12] Zhou Z, Siddiquee MMR, Tajbakhsh N, Liang J, UNet++: (2018) A nested U-Net architecture for medical image segmentation. Deep Learn Med Image Anal Multimodal Learn Clin Decis Support 11045:3–11 10.1007/978-3-030-00889-5_1 PMC7329239 32613207

[13] Lüddecke T, Ecker A (eds) (2022) Image segmentation using text and image prompts. Proceedings of the IEEE/CVF conference on computer vision and pattern recognition

[14] Hu EJ, Shen Y, Wallis P, Allen-Zhu Z, Li Y, Wang S et al (2022) Lora: Low-rank adaptation of large language models. ICLR 1(2):3

[15] Karaev N, Rocco I, Graham B, Neverova N, Vedaldi A, Rupprecht C (2023) CoTracker: It is Better to Track Together. arXiv [csCV]

[16] Campello RJGB, Moulavi D, Sander J (2013) Density-based clustering based on hierarchical density estimates. Advances in Knowledge Discovery and Data Mining. Lecture notes in computer science. Springer Berlin Heidelberg, Berlin, Heidelberg, pp 160–172

[17] Ester M, Kriegel H, Sander J, Xu X (1996) A density-based algorithm for discovering clusters in large spatial databases with noise. KDD. :226–231

[18] Picozzi P, Nocco U, Labate C, Gambini I, Puleo G, Silvi F et al (2024) Advances in Robotic Surgery: A Review of New Surgical Platforms. Electronics 13(23):4675

